# Annotations

**Published:** 1888-08-04

**Authors:** 


### Annotations.

**Paupers for Farmers.**

If Mr. Herbert Mills were a vain man, he might have felt justified in giving full scope to his vanity on the 23rd ult., when a conference of metropolitan poor-law guardians was held at the board-room of the City of London Union, "to consider the question of the practicability, by means of the poor-law, of adapting workhouse administration in the metropolis and larger centres to the education and training of the able-bodied men in country pursuits, in order to fit them for agricultural work in England and the Colonies" This meeting promises, in fact, the adoption by our boards of guardians of that gospel of Home Colonisation, of which it is Mr. Mills' distinction to have been the apostle. He, appealing to private benevolence for means to start a farm for the poor, could only hope to act on a small scale; the unions, if they so choose, may do it on a large one which would at once relieve the existing pressure on town occupations which causes so much distress. The Rev. S. A. Barnett, who stated the case for agricultural training homes in a reasonable and thoughtful paper, is a man possessed of unsurpassed facilities for studying the condition of the London poor, and he made the alarming statement that in East London there are about 314,000 people who may be defined as unemployed, so precarious and insufficient is the work they can obtain. This is in the East End alone, but there are evidences that the condition of the poor in North and South London will soon be equally serious. This is a terrible army of idle and half-starved men, suffering themselves and dangerous to others; for it is a very wise proverb that says, "A hungry man is an angry man." When the hunger is permanent, the wrath gathers force. Everyone is beginning to feel more or less strongly that "something must be done;" and even those who are most convinced that the unemployed are hopelessly vicious and lazy—certainly, idleness has always had the credit of developing vice and laziness—might well do what they can to forward the experiment suggested by Mr. Mills and Mr. Barnett, even though they accept it merely as a *pis aller*. In one respect we consider the original scheme of Home Colonisation, of which we gave a detailed account in our issue of April 28th (Vol. IV., page 57), superior to Mr. Barnett's. In Mr. Mills' plan, agriculture was not to be the only thing practised in the colony: the ploughman needs food, clothing, conveyance for the produce of the soil; and these things were to be provided for him by fellow colonists, instead of being brought from outside. Also Mr. Mills provided in his scheme work and a place for women; families were not to be broken up; while Mr. Barnett advises that "admission should only be offered to men for whose wives and families support was assured in town." We think this separation of husband and wife unwholesome and mischievous. In preserving the sanctity of the home lies the safety of the nation. "Women and children are perpetual Messiahs," says Bronson Alcott, and the chief fault we find with the scheme as proposed to the metropolitan guardians is the removal of the husband and father—the "head of the house," even if the house is only a garret—from those for whom, more than for himself, he ought to feel he is labouring.

**Cottage Industries in Ireland.**

Whatever views we may hold on the means by which it is to be attained, there can be no doubt that the British nation as a whole is desirous of seeing Ireland prosperous and contented. Separatists and Unionists are so far one in aim, however they may differ in the politics of the question. Perhaps both are at fault in making it so much a question of politics. The lesson that is being to-day most strongly forced on the consciousness of philanthropists is the value of individual effort. United effort is necessary, too; but the one does not preclude the other. Let us, by Act of Parliament and subscribed capital, found a reservoir company to supply a city, but do not let us meanwhile withhold the necessary cup of cold water from our thirsty neighbour, and tell him to wait till the pipes are laid and the water-rate fixed. True charity is eminently practical, and while the politicians have been wrangling about the best means of setting Ireland free, Mrs. Ernest Hart, Mr. Marcus Ward, and others have been trying in modest ways and in neglected corners to make it prosperous. Very modest the methods must be at first, for the inhabitants of these barren districts, who have lived for generations under the shadow of famine, have neither skill nor means to take up great industries. They are all agriculturists, in a region where the land consists pretty equally of bog and granite, and where the potato crop fails, on an average, every seven years. The sea is teeming with fish at their feet, but they have no boats in which they dare venture five miles from shore, or go out at all in rough weather. The best that could be done at present to develop the fishing industry by the most benevolent would be to send some of the most intelligent young men from the poor districts of Connemara to the school established by the Baroness Burdett-Coutts at Baltimore, county Cork, where good work has been done, and the condition of the peasantry much improved. On their return they would be able to manage the large fishing boats, which it would not now be safe to entrust to their inexperienced hands. Meanwhile, an effort is being made to teach the people such arts as they can follow at home—knitting, lace-making, and wood-carving, for the two last of which the Celtic intelligence, naturally artistic, is well-fitted. For this purpose Mrs. Ernest Hart wishes to raise a fund of £200 as a gift, and £1,000 as a loan, to provide teachers and materials for these cottage industries. It is not a large sum; England has often given more to a worse cause. Surely it will soon be raised.

**The Progress of Self-help among Workmen.**

There is a large increase in the Hospital Saturday Fund collections this year, amounting to £500, or about a twenty-fourth part of the whole. Perhaps this journal may claim a little credit for this improvement. It has persistently affirmed the necessity for bringing the claims of hospitals systematically before the working classes, and has insisted that measures should be taken to secure regular contributions, of however small an amount. A few weeks ago Mr. A. W. Webster, of Sunderland—perhaps the oldest and most successful organiser of working-man power in the United Kingdom—addressed the public through our columns. Some little time previously, Mr. Webster related his experience, and promulgated his views in a series of articles in The Hospital from week to week. Our readers among the working class, we are proud to know, are numbered by thousands, and by none is the journal more highly valued. We are sincerely glad to record so improved a result of the Saturday collection. But gratification is not always equivalent to satisfaction. It cannot be said that London yet does its best, or even well on Hospital Saturday. The amount collected, though positively large, is relatively small—very small indeed. It should be at least ten times more than it is, and might be ten times more without putting any strain whatever on the working classes. The efforts now made are annual; they should be weekly. Of course, the workshop collections are continued until September. They should be systematically made all the year round. The Catholics, the Wesleyans, and other religious organisations know the power of the weekly penny. Hospital managers should take an example from them. As a matter of fact, hospital managers display very little interest either in the Saturday or Sunday Fund. They take care to prefer their claim at the proper time, but they do very little else. There is no doubt that if the hospitals would unitedly take up the Saturday and Sunday Funds, far more satisfactory results would be accomplished. The workmen are willing and even anxious to help the hospitals, but their desire is largely frittered away in small and comparatively resultless efforts for want of more and better organisation.

